# IL-17/Th17 Pathway Is Activated in Acne Lesions

**DOI:** 10.1371/journal.pone.0105238

**Published:** 2014-08-25

**Authors:** Hanna-Leena Kelhälä, Riitta Palatsi, Nanna Fyhrquist, Sari Lehtimäki, Juha P. Väyrynen, Matti Kallioinen, Minna E. Kubin, Dario Greco, Kaisa Tasanen, Harri Alenius, Beatrice Bertino, Isabelle Carlavan, Bruno Mehul, Sophie Déret, Pascale Reiniche, Philippe Martel, Carine Marty, Ulrike Blume-Peytavi, Johannes J. Voegel, Antti Lauerma

**Affiliations:** 1 Department of Dermatology, University of Oulu and Medical Research Center, Oulu University Hospital, Oulu, Finland; 2 Unit of Systems Toxicology, Finnish Institute of Occupational Health, Helsinki, Finland; 3 Department of Pathology, University of Oulu and Oulu University Hospital, Oulu, Finland; 4 Research, Galderma R&D, Sophia Antipolis, France; 5 Early Development, Galderma R&D, Sophia Antipolis, France; 6 Department of Dermatology and Allergy, Charite Universitätsmedizin, Berlin, Germany; 7 Department of Dermatology, University of Helsinki and Helsinki University Central Hospital, Helsinki, Finland; INSERM-Université Paris-Sud, France

## Abstract

The mechanisms of inflammation in acne are currently subject of intense investigation. This study focused on the activation of adaptive and innate immunity in clinically early visible inflamed acne lesions and was performed in two independent patient populations. Biopsies were collected from lesional and non-lesional skin of acne patients. Using Affymetrix Genechips, we observed significant elevation of the signature cytokines of the Th17 lineage in acne lesions compared to non-lesional skin. The increased expression of IL-17 was confirmed at the RNA and also protein level with real-time PCR (RT-PCR) and Luminex technology. Cytokines involved in Th17 lineage differentiation (IL-1β, IL-6, TGF-β, IL23p19) were remarkably induced at the RNA level. In addition, proinflammatory cytokines and chemokines (TNF-α, IL-8, CSF2 and CCL20), Th1 markers (IL12p40, CXCR3, T-bet, IFN-γ), T regulatory cell markers (Foxp3, IL-10, TGF-β) and IL-17 related antimicrobial peptides (S100A7, S100A9, lipocalin, hBD2, hBD3, hCAP18) were induced. Importantly, immunohistochemistry revealed significantly increased numbers of IL-17A positive T cells and CD83 dendritic cells in the acne lesions. In summary our results demonstrate the presence of IL-17A positive T cells and the activation of Th17-related cytokines in acne lesions, indicating that the Th17 pathway is activated and may play a pivotal role in the disease process, possibly offering new targets of therapy.

## Introduction

Acne is a common disease characterized by androgen dependence, follicular hyperkeratosis, increased sebum excretion, colonization with *Propionibacterium acnes (P. acnes)* and inflammation. The earliest subclinical acne lesion is the microcomedo, which results from increased proliferation and retention of infundibular keratinocytes [1]. The cytokine IL-1α may have a role in the initiation of microcomedos, by its ability to induce hypercornification of keratinocytes [2], [3]. The formation of microcomedos is preceded by mononuclear infiltrates consisting mainly of CD4+ T-cells and CD68+ macrophages [3]. CD4+ T-cells are the major leukocytes in the early (6–72 h) inflammatory infiltrates in acne lesions, with a small portion of CD1+ dendritic cells. Neutrophils emerge increasingly numerous in the 24 h and 72 h lesions, which are then clinically classified as pustules. At later time points CD8^+^ cells infiltrate in the lesions [4], [5].

It has been suggested that *P. acnes* is involved in the triggering of inflammatory acne via Toll-like receptors (TLRs). The importance of TLR-mediated immune response is supported by the presence of TLR2 expressing cells in inflammatory acne lesions. Furthermore, non-immune cells like keratinocytes and sebocytes express functional TLR2 [6]–[12]. *P. acnes* is considered to be a trigger of exaggerated TLR2 mediated immune responses in acne [13]. TLR2 receptors are involved in the recognition of wide array of microbial molecules mainly in gram-positive bacteria, and also yeasts [14]. Recently, *P. acnes* was shown to activate Nod-like receptor 3 (NLRP3) inflammasome in monocytic cells leading to the production of IL-1β [15], [16]. However, it is still unclear whether *P. acnes* can initiate comedogenesis or early phase inflammatory reaction in acne [17]. Also other triggers than *P. acnes* for the early inflammatory cascades in acne lesion formation for example leukotriens or free fatty acids should be considered [18].

In addition to innate immunity, also adaptive immunity, and especially the Th17 pathway, may contribute significantly to the inflammatory response in acne [18], [19]. Previously *P. acnes* has been shown to stimulate the production of IL-17A and IFN-γ in peripheral blood mononuclear cells (PBMCs) [20]–[22]. Moreover, the increased expression of cytokines and other inflammatory markers such as IL-1α, beta-defensins 1 and 2, TNF-α, IL-1β, IL-8, IL-10, matrix metalloproteinases MMP-1, MMP-3, MMP-9, CXCL-2 was found in acne lesions in vivo [3], [23]–[26].

This study was based on the analyses of skin biopsies from clinically early looking inflamed acne lesions (comedones with minimal erythematosus flare or small papules). The study material was recruited by two clinical centers - Oulu, Finland and Berlin, Germany - with independent groups of patients with acne vulgaris, as well as psoriasis patients and healthy volunteers as controls. Our results show that, as in psoriasis, the Th17 pathway is significantly up-regulated both at the RNA and protein level in lesions of acne vulgaris. The results suggest a novel pathomechanism in inflammatory acne, and open up the possibility for a new class of therapeutics targeting the Th17 system in severe acne.

## Materials and Methods

### Ethics statement

The studies presented in this manuscript have been approved by local Ethics committees of Oulu University Hospital in Finland and the Charite Universitätsmedizin Berlin in Germany. The biopsies were taken with informed and written consent. All clinical investigations were conducted in accordance with the Declaration of Helsinki Principles.

### Subjects and sampling

**Figure 1:**
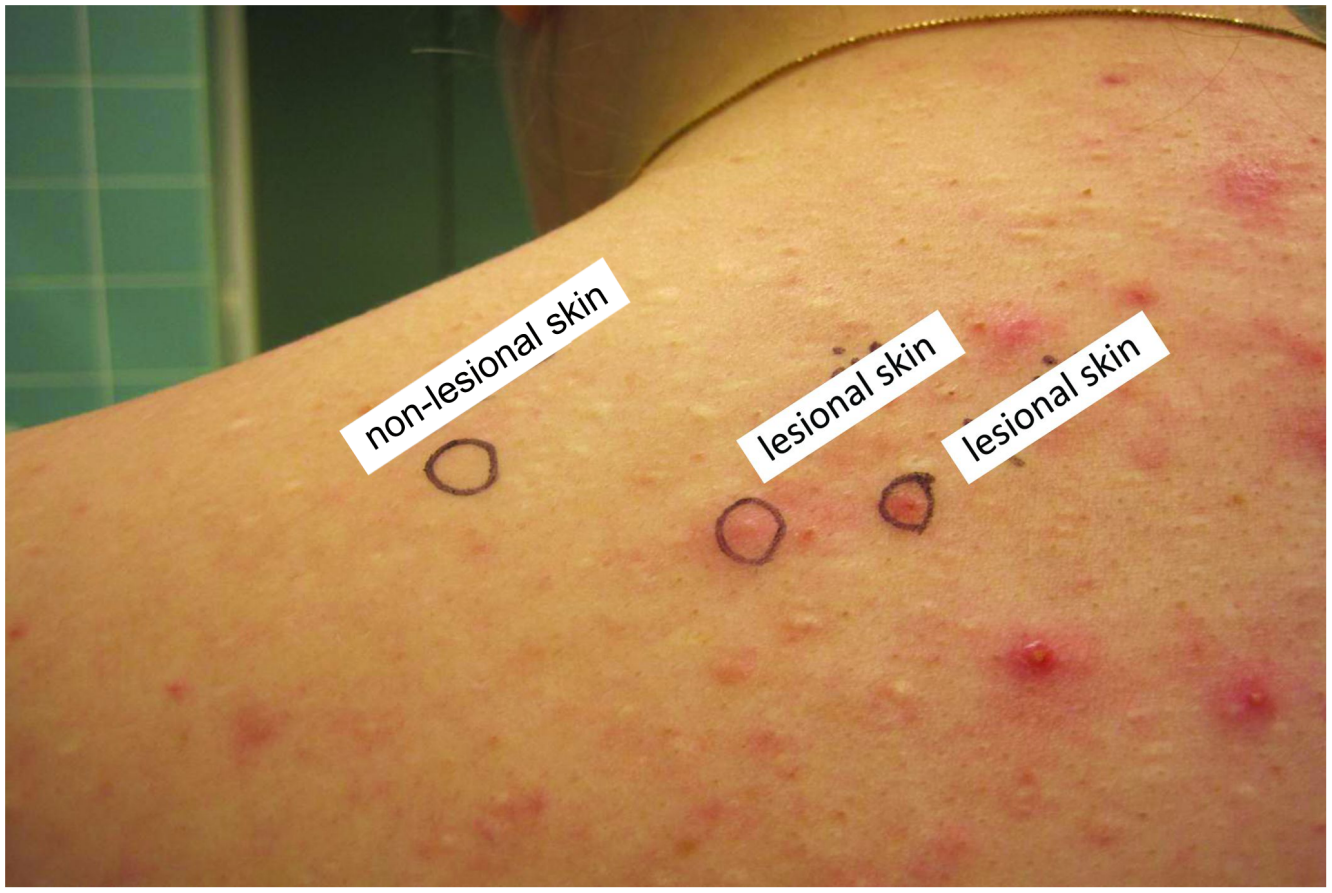
Macroscopic appearance of biopsied lesions from acne patients.

The study was performed in two clinical centers in Oulu, Finland and Berlin, Germany. A total of 56 acne patients with moderate to severe acne vulgaris were included in the study. Clinical characteristic of patients is presented in Tables 1 and 2. Control subjects comprised patients with psoriasis (n = 9, age range 28–65; mean 52.9, not age-matched) and healthy volunteers that had never had acne (n = 6, age range 23–36; mean 26.5). The inclusion criteria for psoriasis patients were chronic plaque psoriasis, not receiving systemic or UV-treatment for at least 1 month before sampling. 3 mm (German cohort) or 4 mm (Finnish cohort) punch biopsies were obtained under local anesthesia from the back or chest of acne patients from lesional and non-lesional skin. The acne lesions obtained by biopsy were identified clinically as early inflammatory acne lesions meaning comedones which were shading into small red papules (Figure 1). The control skin biopsies were taken from the back of psoriasis patients lesional and non-lesional skin and healthy volunteers back skin. Utilization of all biopsies is described in Figure S1.

**Table 1:** Clinical characteristics of acne patient group from Finland.

Finnish patients (n = 20)				
Subject	Age^1^	Gender	Acne severity^2^	Technology^3^
1	21	female	2	RT-PCR and immunohistochemistry
2	24	female	1	
3	21	female	2	
4	17	female	3	
5	34	female	1	
6	25	female	1	
7	29	female	2	
8	22	male	3	
9	29	male	3	
10	21	male	2	
11	26	female	2	
12	22	male	3	
13	19	male	2	
14	27	male	3	
15	25	male	1	
16	26	male	2	
17	25	male	4	
18	36	female	1	
19	31	female	2	
20	18	female	2	
**Mean**	**24.9±5.1**			

1 Data are expressed as mean ±SD.

2 Acne severity at the biopsy site (back or chest) was assessed by Leeds revised acne grading system in Finnish patient group.

3 The technology, which was used to investigate the biopsies taken from the patients.

**Table 2:** Clinical characteristics of acne patient group from Germany.

German patients (n = 36)				
Subject	Age^1^	Gender	Acne severity^2^	Technology^3^
1	18	male	Severe	Affymetrix and RT-PCR
2	19	male	Severe	
3	20	female	Moderate	
4	21	male	Severe	
5	28	male	Moderate	
6	26	male	Severe	
7	22	male	Severe	
8	30	male	Mild	
9	23	male	Severe	
10	24	male	Moderate	
11	19	male	Severe	
12	26	male	Mild	
13	25	male	Moderate	Luminex
14	20	male	Severe	
15	28	male	Moderate	
16	27	male	Moderate	
17	21	male	Moderate	
18	22	male	Mild	
19	25	male	Moderate	
20	19	male	Severe	
21	20	male	Mild	
22	29	female	Moderate	
23	31	male	Severe	
24	31	male	Moderate	
25	18	male	Severe	Immunohistochemistry
26	19	male	Moderate	
27	29	male	Severe	
28	24	male	Severe	
29	30	male	Severe	
30	20	male	Moderate	
31	19	male	Moderate	
32	19	female	Moderate	
33	27	female	Severe	
34	29	male	Moderate	
35	22	male	Severe	
36	21	male	Moderate	
**Mean**	**23.6±4.2**			

1 Data are expressed as mean ±SD.

2 Acne severity at the biopsy site (back) was assessed by Global Evaluation Acne scale in German patient group.

3 The technology, which was used to investigate the biopsies taken from the patients.

### Affymetrix

Biopsies for the microarray analysis were stored in RNA Stabilization Reagent. For RNA extraction the samples were homogenized with a potter in Qiagen lysis buffer. Total RNA was extracted using miRNeasy extraction kits according to manufacturer's protocol. RNA quantity was measured using Nanodrop spectrophotometer ND8000. RNA quality was monitored using a 2100 Bioanalyzer (Agilent Technologies, Waldbronn, Germany). Probes were synthesised and then hybridized on Affymetrix U133 Plus 2.0 chips (Affymetrix, Santa Clara, CA). All chips were normalized using RMA method [27]. Only Affymetrix identifiers (IDs) with expression ≥2exp6 in at least one condition were selected. Finally, 38014 out of 54675 initially present IDs were selected. Data analysis was performed on Array Studio software (OmicSoft, Cary, NC). Mean expression levels were obtained by calculating the geometrical means of the RMA-normalized data for involved and non-involved sample groups, respectively. A two sided paired T test was performed using Array Studio (OmicSoft Corporation, USA), to determine which genes were significantly differentially expressed between involved and non-involved groups, and Benjamini-Hochberg false discovery rate (FDR) multiple testing correction [28] was applied. The raw data is available at NCBI GEO, accession number GSE53795.

### RT-PCR analysis

Snap frozen skin biopsies were homogenized in Trisure (Bioline, London, UK) and RNA was extracted according to standard protocols and subjected for cDNA synthesis. Complementary DNA synthesis, and RT-PCR analysis were performed as previously described [29] with 7500 Fast Real Time PCR-system (Applied Biosystems, Foster City, CA) in Finland and Applied Biosystems 7900HT machine in France. The data were analysed by using the 2^−(ΔΔCt)^ method, according to the manufacturer's instructions. Statistical analyzes were done with Student's t-test or when variances were significantly different, with nonparametric Mann-Whitney U test using GraphPad Prism software (GraphPad Software Inc., LaJolla, CA).

### Cytokine profiling by Luminex technology

Cytokines were extracted from non-lesional and lesional skin which were crushed manually for 4 minutes with a glass potter (Dominique Dutscher S.A, Brumath, France) in 200 µL ice-cold PBS containing Triton X100 0, 2% and protease inhibitors (cOmplete, Mini, EDTA-free) (Roche Applied Science, Mannheim, Germany). Samples were stirred for 30 minutes at 4 °, at 1400 RPM and then centrifuged for 5 minutes at 4°C, at 10 000 RPM. Supernatants were harvested and the concentration of proteins was determined by colorimetric method (Bio-Rad DC Kit)(Bio-Rad Laboratories, Hercules CA). Cytokines were quantified in duplicate using following Luminex assays (Life Technologies, Carlsbad, CA): Human Cytokine, Premixed 23 Plex, Immunoassay Procarta kit and Milliplex Human Cytokine, Premixed 42 Plex, Immunoassay kit (Merck Millipore, Billerica, MA). Cytokine quantitities were normalized to the total concentration of protein.

### Histology and immunohistochemistry

Biopsies for histological analysis were fixed in 10% buffered formaldehyde. After dehydration/impregnation and inclusion in paraffin, 4 µm-thick serial sections were prepared. Slides were stained using Hematoxylin-Eosin.

For immunohistochemistry of German patient samples, deparaffinised sections were treated for antigen retrieval in 10 mM citrate buffer, pH 6.0 at 98°C for 20 minutes. Mouse antihuman CD3 (clone Ab-9, Thermo Scientific, Fremond, CA) was used to detect T-lymphocytes. IL-17A was detected on the same section with a goat polyclonal antihuman IL-17A antibody (R&D systems, Minneapolis, MN). CD3 was detected with Alexa fluor 594 conjugated antibody and IL-17A was revealed after amplification (Biotin/ Streptavidin FITC). Positive cells were counted visually on slides scanned with the NanoZoomer (Hamamatsu, Japan).

For immunohistochemistry of Finnish samples, heat-induced antigen retrieval of the deparaffinised sections was performed in a microwave oven. Endogenous peroxidase activity was neutralized. The sections were incubated with monoclonal mouse anti-human antibodies to CD4 (clone 4B12, 1∶50, Novocastra, Newcastle, UK), CD8 (clone 4B11, 1∶200, Novocastra), CD68 (clone KP-1, 1∶10000, Dako, Copenhagen, Denmark), CD83 (clone 1H4b, 1∶25, Dako), Foxp3 (clone 236A/E7, 1∶100, Abcam Ltd., Cambridge, UK), T-bet (clone 4B10, 1∶200, Abcam Ltd.) and goat polyclonal anti-human antibody to IL-17A (1∶100, R&D systems, Minneapolis, MN). Bound antibodies were detected using the NovoLink Polymer detection system (Leica Biosystems, Newcastle, UK), the EnVision system (Dako) or Goat-HPR-Polymer kit (Biocare Medical, Concord, CA). Deaminobenzidine (DAB) was used as the chromogen and hematoxylin as the counterstain. Digital image analysis was used to count the positive cells. The digital images were obtained under 20× magnification from all sections with the use of a Nikon Eclipse E600 microscope and a Olympus DP25 digital camera. Images were taken from upper dermal area and around pilosebaceous follicles, resulting in 2 to 7 images per slide. Image analysis was performed as described previously using ImageJ software [30] to record the average number of the positively stained cells per 20× field image (0.14 mm^2^). Data are presented as median positive cell count (interquartile range). The statistical analysis was performed by IBM SPSS Statistics 19 (IBM, Chicago, IL). Mann-Whitney U test two-tailed p-value less than 0.05 was considered statistically significant.

## Results

### Genome-wide transcriptome reveals Th17-related signaling significantly elevated in acne lesions

**Figure 2:**
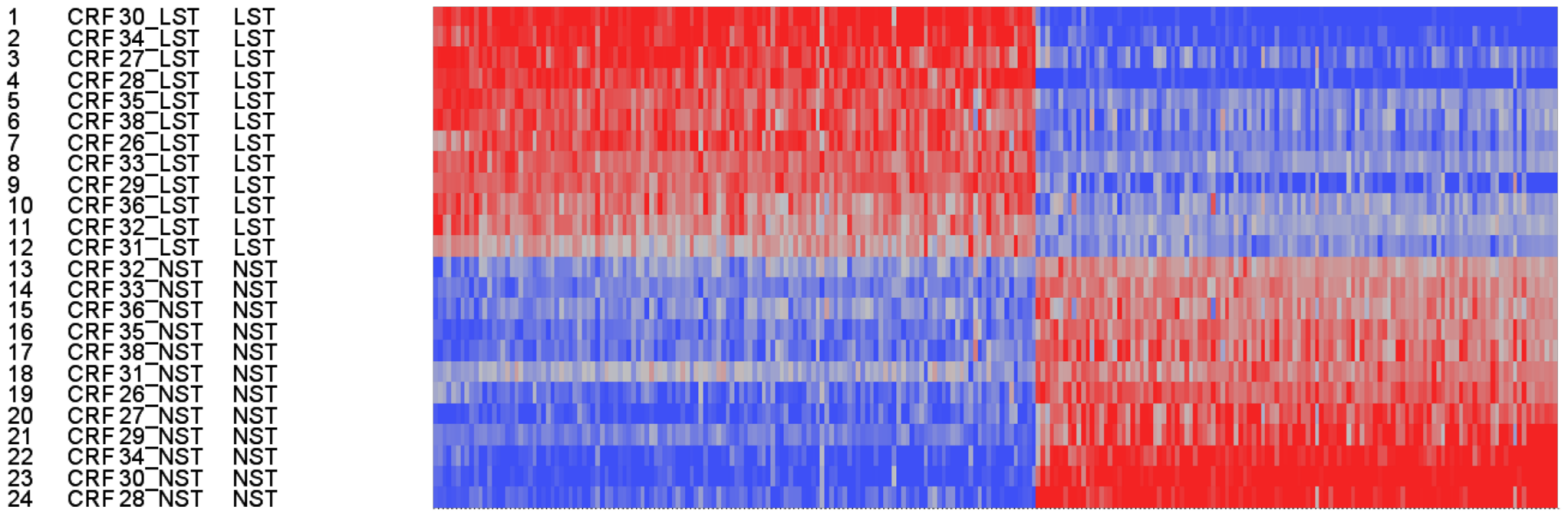
2-components NMF (Non Negative Matrix Factorization) model. The heatmap of 904 regulated genes on subjects-corrected log2-expressions (legend color blue for low expression and red for high expression).

**Figure 3:**
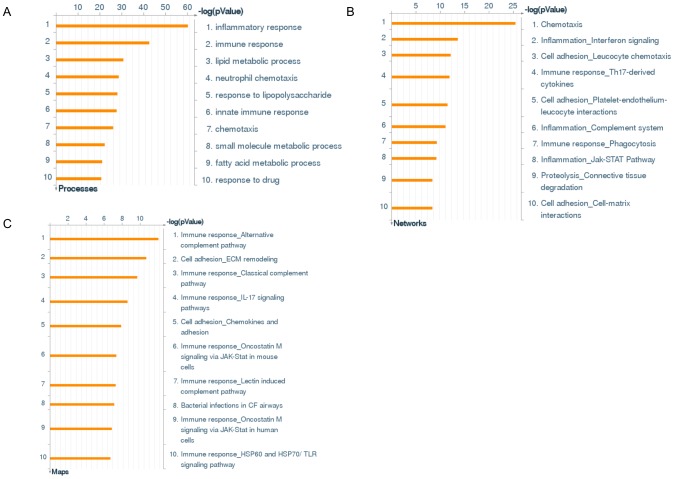
Bioinformatic analysis of differentially modulated genes. Gene enrichment analysis in three different functional ontologies were performed using MetaCore: biological processes (a), process networks (b) and canonical pathway maps (c).

The study was performed in two separate patient cohorts from Germany and Finland (Tables 1 and 2). The gene expression in skin biopsies of the subjects recruited in Germany was screened using Affymetrix microarray technology. In the array analyses, a total of 904 genes were differentially expressed in acne lesions compared with non-lesional skin, and 509 of these were upregulated, whereas 395 were downregulated (Tables 3 and 4). All microarray data have been deposited in NCBI's Gene Expression Omnibus [31] and are accessible through GEO series accession number GSE53795. A two-component Non Negative Matrix Factorization (NMF) model [32] was applied and allowed a clear separation between lesional and non-lesional acne samples (Figure 2). Bioinformatic analysis of differentially modulated genes using Metacore software identified the inflammatory and immune responses as the main enriched biological processes (Figure 3a). Interestingly, the IL-17 signaling pathway and the Th17-derived cytokines network were ranked fourth in the enriched GeneGo pathways and networks, respectively (Figures 3b and 3c).

**Table 3:** Top 20 up-regulated genes in the German acne patient group based on Affymetrix microarray technology.

ProbeSet	Gene symbol	Title	Mean expression (non-involved skin)	Fold change (involved vs. non-involved skin)	FDR
232170_at	S100A7A	S100 calcium binding protein A7A	235	29	<0.001
202859_x_at	IL8	interleukin 8	162	25	<0.001
207356_at	DEFB4	defensin, beta 4	451	25	<0.001
211906_s_at	SERPINB4	serpin peptidase inhibitor, clade B (ovalbumin), member 4	768	19	<0.001
204475_at	MMP1	matrix metallopeptidase 1 (interstitial collagenase)	124	19	<0.001
212768_s_at	OLFM4	olfactomedin 4	65	16	0.0018
205828_at	MMP3	matrix metallopeptidase 3 (stromelysin 1, progelatinase)	79	16	<0.001
204470_at	CXCL1	chemokine (C-X-C motif) ligand 1 (melanoma growth stimulating activity, alpha)	51	15	<0.001
210873_x_at	APOBEC3A	apolipoprotein B mRNA editing enzyme, catalytic polypeptide-like 3A	53	14	<0.001
205863_at	S100A12	S100 calcium binding protein A12	47	14	<0.001
203691_at	PI3	peptidase inhibitor 3, skin-derived	987	13	<0.001
209875_s_at	SPP1	secreted phosphoprotein 1	96	13	<0.001
206561_s_at	AKR1B10	aldo-keto reductase family 1, member B10 (aldose reductase)	135	13	<0.001
205681_at	BCL2A1	BCL2-related protein A1	81	13	<0.001
206336_at	CXCL6	chemokine (C-X-C motif) ligand 6 (granulocyte chemotactic protein 2)	13	11	<0.001
209774_x_at	CXCL2	chemokine (C-X-C motif) ligand 2	54	11	<0.001
204580_at	MMP12	matrix metallopeptidase 12 (macrophage elastase)	67	11	<0.001
205114_s_at	CCL3/CCL3L1/CCL3L3	chemokine (C-C motif) ligand 3/ chemokine (C-C motif) ligand 3-like 1/chemokine (C-C motif) ligand 3-like 3	65	10	<0.001
210146_x_at	LILRB2	leukocyte immunoglobulin-like receptor, subfamily B (with TM and ITIM domains), member 2	32	10	<0.001
210413_x_at	SERPINB3/SERPINB4	serpin peptidase inhibitor, clade B (ovalbumin), member 3/ serpin peptidase inhibitor, clade B (ovalbumin), member 4	1936	10	<0.001

**Table 4:** Top 20 down-regulated genes in the German acne patient group based on Affymetrix microarray technology.

ProbeSet	Gene symbol	Title	Mean expression (non-involved skin)	Fold change (involved vs. non-involved skin)	FDR
204515_at	HSD3B1	hydroxy-delta-5-steroid dehydrogenase, 3 beta- and steroid delta-isomerase 1	2897	−8.8	0.002
205380_at	PDZK1	PDZ domain containing 1	1914	−6.4	0.002
210576_at	CYP4F8	cytochrome P450, family 4, subfamily F, polypeptide 8	1305	−6.3	0.002
241412_at	BTC	betacellulin	791	−6.1	<0.001
234513_at	ELOVL3	elongation of very long chain fatty acids (FEN1/Elo2, SUR4/Elo3, yeast)-like 3	8094	−6.1	0.003
1553583_a_at	THRSP	thyroid hormone responsive (SPOT14 homolog, rat)	5008	−5.9	0.003
206143_at	SLC26A3	solute carrier family 26, member 3	166	−5.7	<0.001
234980_at	TMEM56	transmembrane protein 56	3401	−5.4	0.002
220801_s_at	HAO2	hydroxyacid oxidase 2 (long chain)	1120	−5.3	<0.001
230573_at	SGK2	Serum/glucocorticoid regulated kinase 2	495	−5.3	0.0016
207958_at	UGT2A1/UGT2A2	UDP glucuronosyltransferase 2 family, polypeptide A1/UDP glucuronosyltransferase 2 family, polypeptide A2	351	−5.0	<0.001
206465_at	ACSBG1	acyl-CoA synthetase bubblegum family member 1	8201	−4.9	0.002
208962_s_at	FADS1	fatty acid desaturase 1	5240	−4.8	0.003
205029_s_at	FABP7	fatty acid binding protein 7, brain	4530	−4.8	0.011
239108_at	FAR2	Fatty acyl CoA reductase 2	6891	−4.7	0.002
210377_at	ACSM3	acyl-CoA synthetase medium-chain family member 3	591	−4.5	0.003
206714_at	ALOX15B	arachidonate 15-lipoxygenase, type B	6853	−4.5	0.003
221142_s_at	PECR	peroxisomal trans-2-enoyl-CoA reductase	1668	−4.5	0.0012
236175_at	TRIM55	tripartite motif-containing 55	184	−4.4	<0.001
208331_at	BPY2	basic charge, Y-linked, 2	1084	−4.4	0.007

**Figure 4:**
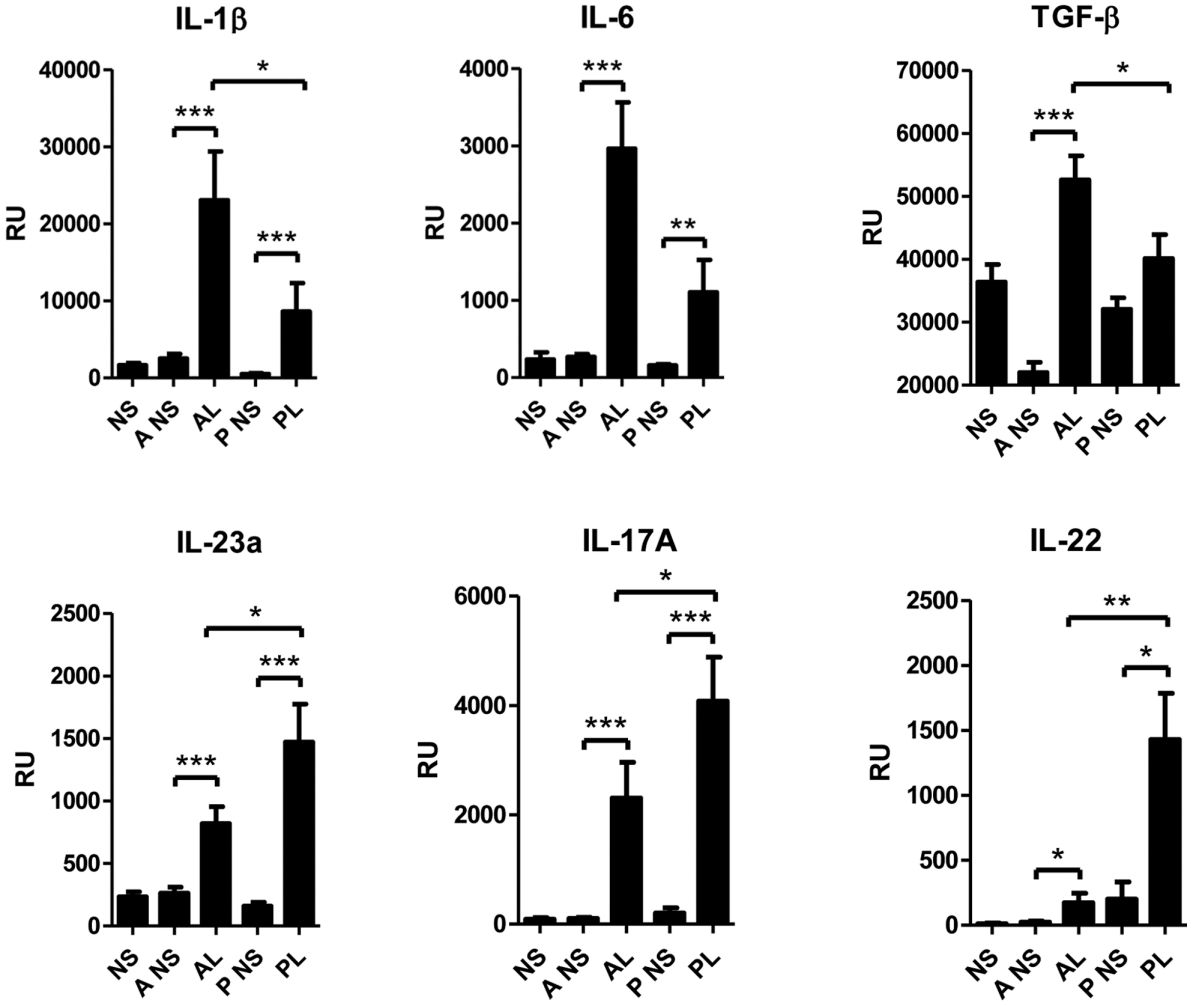
Th17 related cytokines were increased in acne lesions. The skin biopsies were obtained from healthy volunteers normal skin (NS), from acne patients non-lesional skin (A NS) and lesional skin (AL) and from psoriasis patients non-lesional skin (P NS) and lesional skin (PL). The expression levels of cytokines were determined by RT-PCR. The expressions of inflammatory cytokines TGF-β, IL-6, IL-1β and IL-23 were increased significantly both in acne and psoriasis but IL-1β and TGF-β expression was higher in acne. IL-23 was increased more in psoriasis lesions. IL-17A was significantly expressed in lesional acne and psoriasis skin. IL-22, was slightly elevated in acne and psoriasis, however significantly more in psoriasis. *P<0.05, **P<0.01, ***P<0.001; bars represent mean +SEM. RU, relative units; TGF-β, transforming growth factor- beta; Th17, T helper type 17.

The Affymetrix transcriptome results were confirmed with real-time PCR (RT-PCR). Among the upregulated genes were IL-23, IL-6 and TGF-β, which all are critically involved in Th17 cell activation and differentiation. Furthermore, Th17 cell products IL-17A, IL-22, IL-26, TNF and IL-17 induced chemokines CSF2 and CCL20 were also expressed at higher levels in acne lesions (Table 5). The expression of the upregulated genes of Th17 lineage inducing cytokines IL-1β, IL-6, TGF-β and IL-23 were found significantly increased also in the Finnish cohort by RT-PCR (Figure 4) as well as Th17 signaling cytokine IL-17A.

**Table 5:** Expression at the mRNA level of markers and cytokines characterizing the Th17 cell subtype in the German acne cohort.

Th17 signalling	Markers and cytokines		Affymetrix data			RT-PCR		
	Gene symbol	Title	Mean expression (non-involved skin)	Fold change (involved vs. non- involved skin)	FDR (involved vs. non- involved skin)	Mean CT (non- involved skin)	Fold change (involved vs. non- involved skin)	FDR (involved vs. non- involved skin)
**Th17 activation**	IL23	interleukin 23, alpha subunit p19	95	1.3	0.001	33	6.2	<0.001
	TGFB1	transforming growth factor, beta 1	111	1.8	<0.001	24	1.1	0.08
	IL6	interleukin 6	73	4.7	0.001	30	6.2	<0.001
	STAT3	signal transducer and activator of transcription 3	962	1.9	0.001	22	-1.4	0.25
**Th17 related cytokines and chemokines**	IL17A	interleukin 17A	13	4.3	0.01	32	5.6	<0.001
	IL17F	interleukin 17F	35	2.5	0.01	Not evaluated		
	IL22	interleukin 22	9	1.8	0.11	34	2.4	<0.001
	IL6	interleukin 6	73	4.7	0.001	30	6.2	<0.001
	TNF	tumor necrosis factor	80	1.6	0.003	28	1.1	0.31
	IL26	interleukin 26	24	2.0	0.002	31	1.5	0.004
	CCL20	chemokine (C-C motif)ligand 20	146	3.3	0.002	29	2.4	<0.001
	IL21	interleukin 21	Not detected			34	1.3	0.14
	CSF2	colony stimulating factor 2	91	1.1	0.06	32	1.3	0.08
**Receptors**	IL23R	interleukin 23 receptor	Not detected			34	−1.6	0.10
	CCR6	chemokine (C-C motif) receptor 6	91	1.2	0.33	28	−1.8	<0.001

Abbreviations: CT, cycle threshold value; FDR, False discovery rate.

**Figure 5:**
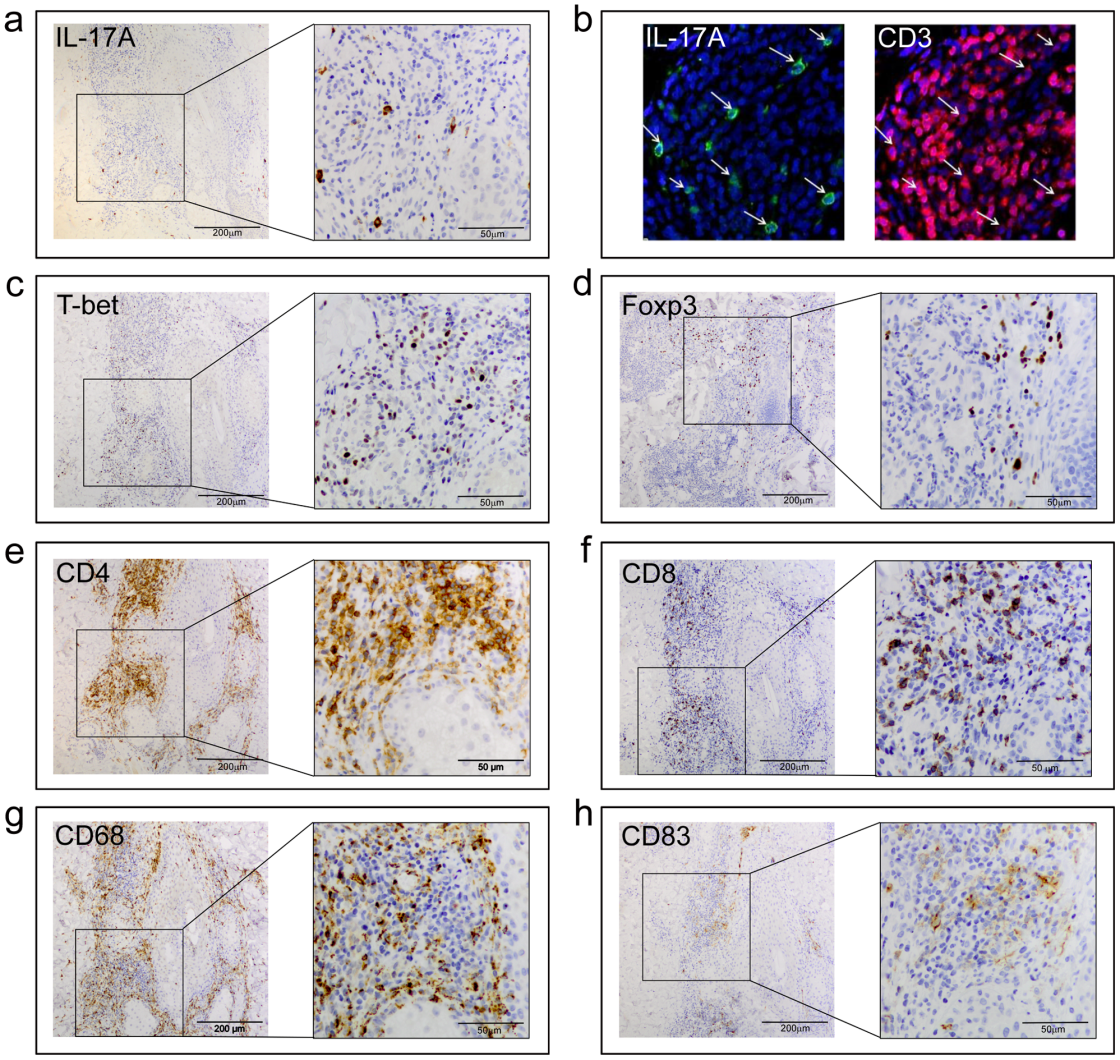
Inflammatory cell infiltrates of early acne lesions in immunohistochemistry. Representative immunohistochemical staining showing IL-17A+ cells in early acne lesion (**a**). Staining of IL-17A+ and CD3+ cells shows that IL-17A (in green) and CD3 (in red) were detected in acne vulgaris lesions. Cell nuclei were counterstained in blue. Arrows indicate IL-17A and CD3 double positive cells (**b**). T-bet+ cells (**c**) were more numerous than IL-17A+ cells or Foxp3+ cells (**d**) in the lesional acne skin. Large number of CD4+ cells (**e**), mostly lymphocytes, was seen around pilosebaceous unit and perivascularly. CD8+ cells (**f**) were fewer in number than CD4+ cells. Large number of CD68+ macrophages (**g**) and a few CD83+ cells (**h**), which are mature dendritic cells, were detected around sebaceous follicles. (Bar = 200 µm; in the insets bar  = 50 µm).

The increased production of Th17 related cytokines in the acne lesions was confirmed at the protein level by cytokine profiling of manually crushed skin biopsies from German patients by Luminex technology (Table 6). Furthermore, significantly higher numbers of IL-17A positive (IL-17A+) cells were detected by immunohistochemistry both in the papillary dermis and around sebaceous follicles in acne lesions of Finnish patients (Table 7). IL-17A+ cells were scattered in the superficial and deep layers of the dermis (Figure 5a). IL-17A+ cells were mainly lymphocytes but some of them were neutrophils and even mast cells (data not shown). IL-17A+ cells were also identified in the inflammatory infiltrates in the German patient group (Figure 5b). Strongly IL17-positive cells were mainly composed of CD3+ lymphocytes in both lesional and non-lesional samples.

**Table 6:** Expression at the protein level of markers and cytokines characterizing the Th17 cell subtype in the German acne cohort.

Th17 Signalling	Symbol	Title	Protein level pg/mg (non-involved skin)	Fold change (involved vs. non- involved skin)	P-value (involved vs. non- involved skin)
**Th17 activation**	IL23	interleukin 23, alpha subunit p19	0.3	3.1	0.17
	IL6	interleukin 6	1.1	15.0	<0.001
**Th17 related cytokines and chemokines**	IL17A	interleukin 17A	1.1	3.8	0.01
	IL17F	interleukin 17F	0.3	4.9	<0.001
	IL22	interleukin 22	4.6	3.1	0.03
	IL6	interleukin 6	1.1	15.0	<0.001
	TNF	transforming growth factor, beta 1	1.1	2.5	0.02
	CCL20	chemokine (C-C motif)ligand 20	0.4	2.6	0.04
	IL21	interleukin 21	7.5	3.9	0.01
	CSF2	colony stimulating factor 2	1.1	2.2	0.01

**Table 7:** Number of positively stained cells in lesional and non-lesional skin of acne patients (n = 16)^1^ as assessed by immunohistochemistry.

	Positive-cell counts^2^ in upper dermis			Positive-cell counts^2^ around follicles		
	Lesional	Non-lesional	P-value	Lesional	Non-lesional	P-value
CD4	33 (22–52.8)	16.5 (9.3–26.3)	**0.007**	52 (44–127.5)	8 (4–14.5)	**6.5×10^−5^**
CD8	6 (2.3–15.3)	3 (1.3–9.5)	**0.225**	11 (4.5–18.5)	1 (1–2)	**8.5×10^−5^**
T-bet	6 (3–17)	2.5 (1–3)	**0.005**	16.5 (3.5–31.5)	0 (0–1.8)	**8.6×10^−5^**
IL–17A	4.5 (2.3–8)	2 (2–3)	**0.030**	6 (3.5–9.3)	3 (1.3–5.3)	**0.023**
Foxp3	4.5 (2–9.8)	1 (1–3)	**0.003**	6 (0.8–13)	1 (1–2)	**0.072**
CD68	38 (18.3–68)	5.5 (1–13)	**3.1×10** **^–5^**	77.5 (27–131.5)	3.5 (2.3–5.8)	**2.6×10^−5^**
CD83	7 (2.3–13)	1.5 (0.3–2.8)	**1.5×10** **^–4^**	12 (4.5–27)	1 (0–2)	**2.3×10^−6^**

Abbreviations: IHC, immunohistochemistry; IQR, interquartile range.

1 16 of total 20 acne patients samples in Finnish patient cohort were examined by IHC.

2 Positive-cell counts per field image are expressed as medians (IQR). Statistically significant difference of P<0.05 in the lesional acne skin compared to non-lesional skin. Significant P values are bold.

Thus, the upregulation of Th17 pathway found with global Affymetrix analysis was confirmed with RT-PCR and also on the protein level with Luminex and immunohistochemistry in patient samples from Germany. Moreover, the result was similar in a separate patient group from Finland investigated by RT-PCR and immunohistochemistry. While IL-17A in addition to Th17 cells can also be secreted by other CD3+ lymphocytes, including CD8+ cytotoxic T-cells (Tc17) and γδ T cells, the concomitantly observed inductions of Th17-activating and Th17-related cytokines and chemokines observed in two independent patient populations support the observation of Th17 pathway activation in acne lesions.

### Innate immune responses were significantly increased in acne lesions

**Figure 6:**
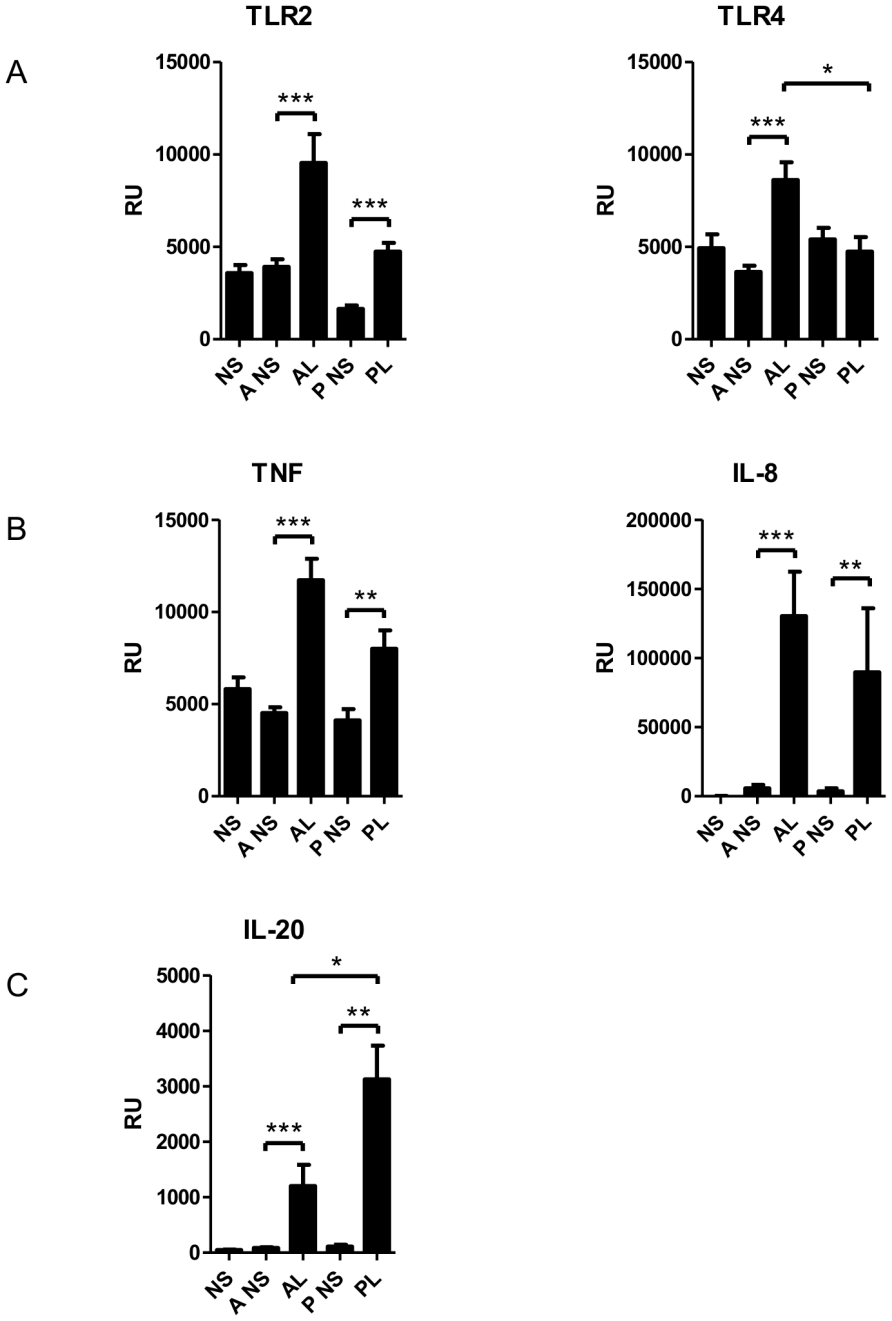
The expression of proinflammatory mediators in Finnish patient cohort examined by RT-PCR. The expression of **a**) toll-like receptors TLR2 and TLR4, **b**) proinflammatory cytokine TNF-α and neutrophil chemotactic factor IL-8 were significantly up-regulated in lesional acne. **c**) IL-20 mRNA were increased in acne and psoriasis lesions. It is known that IL-20 can stimulate epidermal hyperplasia in model systems and is upregulated in psoriasis. *P<0.05, **P<0.01, ***P<0.001; bars represent mean ±SEM.

We wanted to investigate also innate immune responses in acne lesions. *P. acnes* triggers inflammatory responses via TLR2 or Nod-like receptor 3 (NLRP3) -inflammasome, leading to the production of IL-6, and IL-8 by keratinocytes and sebocytes and IL-1β, IL-12 and IL-8 by macrophages and primary monocytes, respectively [15], [16], [33]–[35]. The expression of TLR2 and TLR4 were significantly elevated in the acne lesions in the Finnish group (Figure 6a). Of note, TLR2, but not TLR4, was increased in psoriasis control samples as found earlier by Begon et al [36]. The proinflammatory cytokines IL-1β, IL-6, TNF-α and the chemokine IL-8 were all expressed at a higher level in acne lesions in the Finnish cohort (Figure 4, Figure 6b). Similarly, TLR2 and TLR4 as well as IL-8, IL-6, and TNF-α, were induced in acne lesions in the German group (Table 8).

**Table 8:** Expression at the mRNA level of proinflammatory mediators in the German acne cohort determined by Affymetrix microarray technology.

Gene symbol	Title	Mean expression (non-involved skin)	Fold change (involved vs. non- involved skin)	FDR (involved vs. non- involved skin)
TLR2	toll-like receptor 2	682	1.9	0.006
TLR4	toll-like receptor 4	126	2.1	0.0006
IL6	interleukin 6	73	4.7	0.001
TNF	tumor necrosis factor	80	1.6	0.003
IL8	interleukin 8	162	24.7	0.0001

Abbreviations: FDR, false discovery rate.

**Figure 7:**
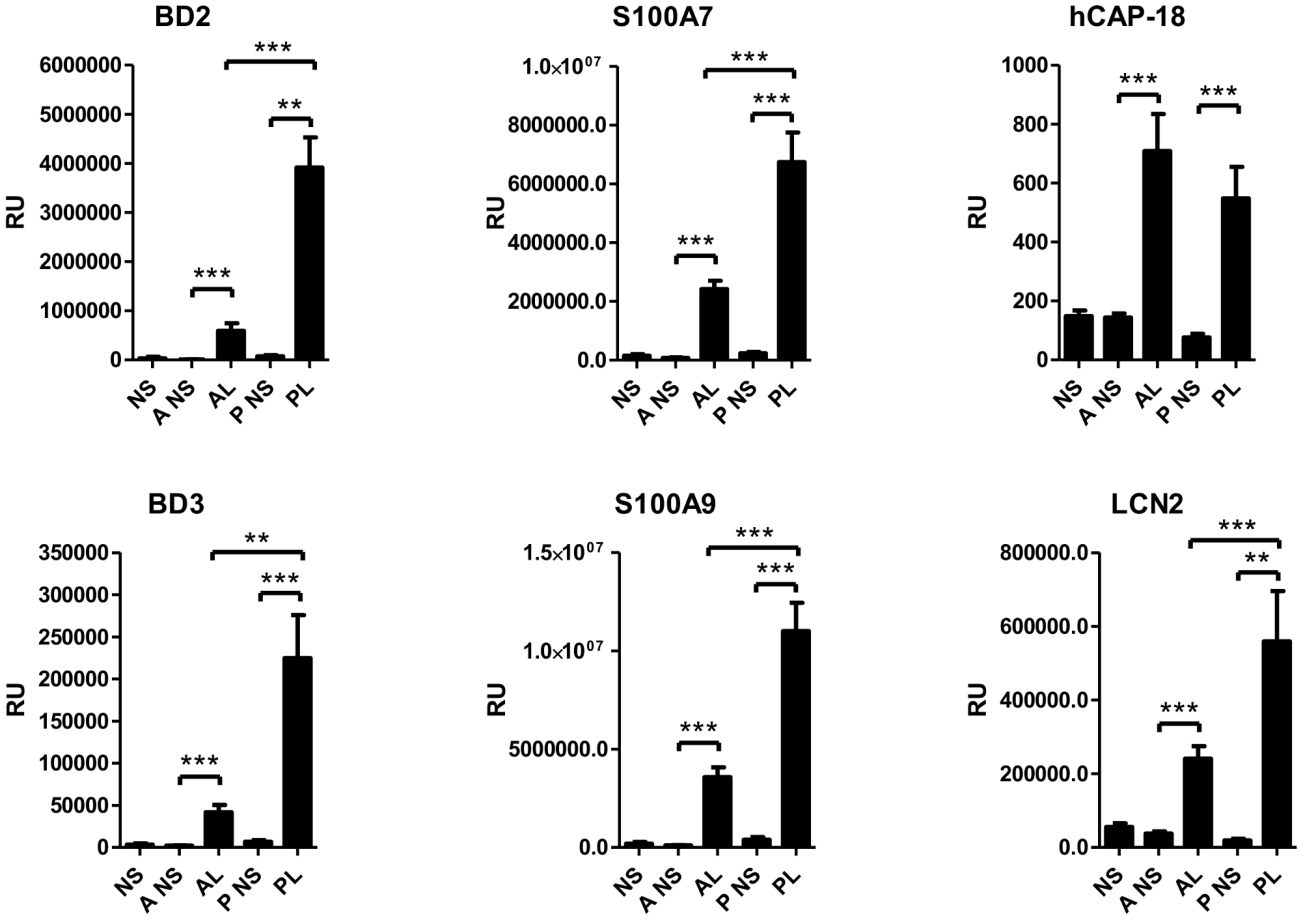
Antimicrobial peptides are increased in acne and psoriasis lesions by RT-PCR. The expressions of mRNAs were significantly higher in psoriasis except for hCAP-18, which was similarly expressed in both. *P<0.05, **P<0.01, ***P<0.001; bars represent mean ±SEM. BD2, human beta-defensin 2; BD3, human beta-defensin 3; hCAP-18, human cathelicidin antimicrobial protein 18; LCN2, lipocalin-2; RU, relative units.

The expression of antimicrobial peptides (AMPs) was also increased in the acne lesions. AMPs are evolutionary conserved, positively charged molecules that bind membranes of microbes by their hydrophobic surfaces and form pores on the membranes and limit the survival of commensals and potential pathogens also with other mechanisms [37]. The expression of AMPs human cathelicidin antimicrobial protein (hCAP18), lipocalin (LCN2), human beta-defensin 2 (hBD2), hBD3, S100A7 and S100A9 were found significantly upregulated in acne lesions in the Finnish patient group by RT-PCR (Figure 7). Similar findings were observed in German patients (Table 9).

**Table 9:** Expression at the mRNA level of antimicrobial peptides in the German acne cohort determined by Affymetrix microarray technology.

Gene symbol	Title	Mean expression (non-involved skin)	Fold change (involved vs. non- involved skin)	FDR (involved vs. non- involved skin)
CAMP	cathelicidin antimicrobial peptide	121	1.5	0.004
DEFB103A	defensin, beta 103B	117	1.9	0.0001
DEFB4A	defensin, beta 4A	451	24.6	0.0001
LCN2	lipocalin 2	1 998	2.6	0.004
S100A7	S100 calcium binding protein A7	7 058	4.1	0.0004
S100A9	S100 calcium binding protein A9	4 086	5.9	0.0003

Abbreviations: FDR, false discovery rate.

### Th1 and T regulatory cell markers were elevated at the RNA level in acne lesions

**Figure 8:**
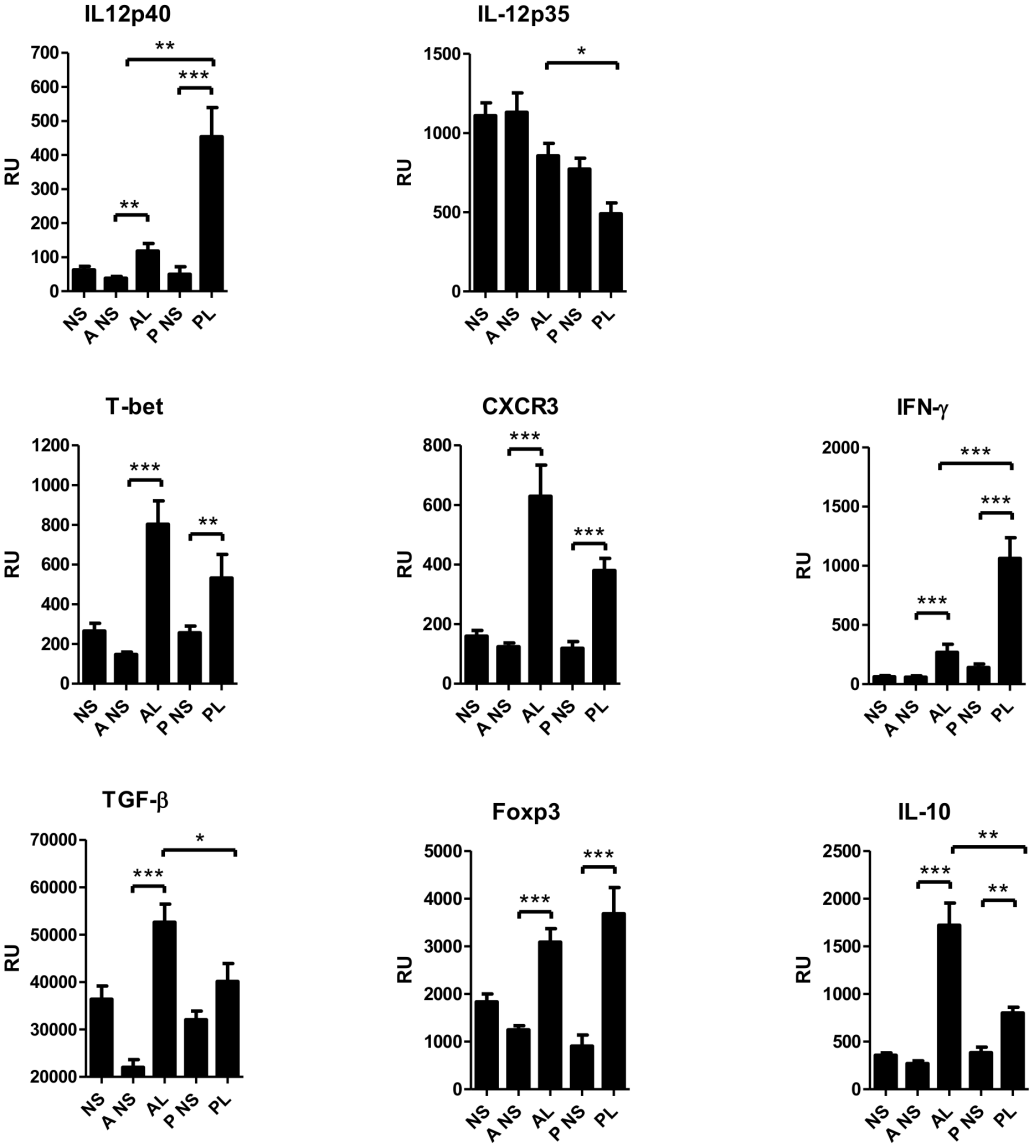
The expression of Th1 cell and Treg markers were activated in acne by RT-PCR. Th1 transcription factor T-bet, Th1 cell surface chemokine receptor CXCR3 and IFN-γ (the effector cytokine of Th1 cells) mRNAs were significantly up-regulated in acne and psoriasis lesions. The transcription factor of Tregs, Foxp3, was elevated in acne and psoriasis. TGF-β which is both Th17 and Treg lineages inducing cytokine was significantly increased in acne lesions. The product of Tregs, immunosuppressive cytokine IL-10 was expressed more in acne than in psoriasis. *P<0.05, **P<0.01, ***P<0.001; bars represent mean ±SEM. RU, relative units; TGF-β, transforming growth factor- beta; Th1, T helper type 1; Treg, regulatory T cell.

In addition to the Th17 pathway, we examined other T-cell subsets in acne lesions. Mouser and co-workers [38] demonstrated a subpopulation of Th1 cells generated from early inflamed acne lesions, but regulatory T cells (Tregs) are not investigated in acne lesions. IL-12 is a pivotal cytokine in activating Th1 responses, and is induced in monocytes by *P. acnes* via TLR2 [6] and in vitro in PBMCs [20]. The IL-12 subunit p40, but not p35, was found significantly increased in both acne (P<0.01) and psoriasis (P<0.001) by RT-PCR (Figure 8). Furthermore, the Th1 polarizing key transcription factor T-bet, Th1 effector cytokine IFN-γ, and Th1 type chemokine receptor CXCR3 were also expressed at a higher level in both acne and psoriasis lesions, indicating the involvement of Th1 effector cells (Figure 8). Similar findings were observed in the German cohort, since STAT1 a positive regulator of Th1 development was induced, and the CXCR3 ligands CXCL9, CXCL10 and CXCL11, were overexpressed in lesional acne skin (Table 10).

**Table 10:** Expression at the mRNA level of markers and cytokines characterizing the Th1 cell subtype in the German acne cohort determined by Affymetrix microarray technology.

Gene symbol	Title	Mean expression (non- involved skin)	Fold change (involved vs. non- involved skin)	FDR (involved vs. non- involved skin)
CXCL10	chemokine (C-X-C motif) ligand 10	131	3.1	0.01
CXCL11	chemokine (C-X-C motif) ligand 11	24	2.8	0.05
CXCL9	chemokine (C-X-C motif) ligand 9	131	2.3	0.04
CXCR3	chemokine (C-X-C motif) receptor 3	86	1.1	0.03
STAT1	signal transducer and activator of transcription 1	500	2.2	0.01
TBX21	T-box 21	65	1.2	0
IL12B	interleukin 12B (p40)	Not detected	NA	NA
IL12A	interleukin 12A (p35)	Not detected	NA	NA
IFNG	interferon, gamma	Not detected	NA	NA

Abbreviations: FDR, false discovery rate; NA, Not applicable.

Foxp3 is a specific marker of natural and induced Tregs, which are efficient suppressors of both innate and adaptive immune responses. TGF-β and IL-10 are involved in the induction and function of Tregs [39]. In the Finnish cohort, the expression of Foxp3, TGF-β and IL-10 were found significantly up-regulated in acne lesions by RT-PCR, and the latter two were at higher levels in acne lesions compared with psoriatic lesions (Figure 8). In the German cohort, TGF-β and IL-10 displayed a slightly enhanced expression in acne lesions and also the expression of Foxp3 was detected (Table 11).

**Table 11:** Expression at the mRNA level of markers and cytokines characterizing the Treg cell subtype in the German acne cohort determined by Affymetrix microarray technology.

Gene symbol	Title	Mean expression (non-involved skin)	Fold change (involved vs. non- involved skin)	FDR (involved vs. non- involved skin)
FOXP3	forkhead box P3	62	1.2	0.002
IL10	interleukin 10	31	1.9	0.002
TGFB1	transforming growth factor, beta 1	111	1.8	0.001

Abbreviations: FDR, false discovery rate.

Th2 cells produced cytokines IL-4, IL-5 and IL-13 were either not detected or not modulated in the German cohort by array technology, and detected at a very low level in the Finnish cohort by RT-PCR, suggesting that Th2 cells are not involved in the inflammatory response in acne and in psoriasis (data not shown).

### Cells of adaptive immunity were recruited to acne lesions

In addition to measurement of gene expression in skin samples, we investigated the infiltration of key cell types in acne lesions. Apart from IL-17A, skin biopsy tissue sections were stained for CD4, CD8, T-bet, Foxp3, CD68 and CD83. All sections of lesional and non-lesional acne samples contained immune cells in papillary dermis and around sebaceous follicles. The most common cells were CD68+ macrophages and CD4+ T-cells. The count of CD4+ cells and CD83+ mature dendritic cells was significantly higher both in the papillary dermis and around follicles in acne lesions, when compared with non-lesional skin. (Figure 5e–h, Table 7). T-bet+ cells were also detected both in lesional and non-lesional skin of acne patients, but at significantly higher numbers in acne lesions, both in the papillary dermis and around follicles, demonstrating the presence of Th1 cells (Figure 5c, Table 7). Yet, the number of Foxp3+ cells was significantly higher in the acne lesions in the papillary dermis but not around follicles (Figure 5d, Table 7). Finally, the count of CD8+ T-cells was significantly higher in infiltrates around follicles in lesional acne (Table 7).

## Discussion

In the current study we examined the inflammatory reaction and especially the IL-23/Th17/IL-17A axis in lesions of acne in vivo. Our results in two independent groups of acne patients show that the cytokines involved in the differentiation of Th17 cells and the expression of the main effector cytokines IL-17A and IL-17F were significantly expressed in lesions of acne at mRNA and protein level.

IL-17A and IL-17F are key cytokines for the recruitment and activation of neutrophils and can target different cell types including keratinocytes, endothelial cells, monocytes, fibroblasts to induce pro-inflammatory mediators IL-6, TNF-α, IL-1β, PGE2, nitric oxide, matrix metalloproteinases and chemokines (GM-CSF, G-CSF, CXCL1, CXCL8, CCL2, CCL7, CCL20) [40]. In psoriasis IL-17A, IL-17F, IL-22 and IL-20 act on keratinocytes leading to epidermal hyperplasia [41], [42]. IL-22, which is secreted by both Th17 and Th22 cells [40], induces the production of IL-20 [43]. In our study elevated levels of IL-22 (Figure 4, Table 5) and IL-20 (Figure 6c) were found in acne lesions by RT-PCR. IL-17 and IL-22 induces the production of AMPs [44]. The expression of AMPs was up-regulated in acne, which confirms prior observations [23], [26], [45]–[48]. Probably due to the high level of AMPs, acne and psoriasis are seldom complicated by secondary infections.

**Figure 9:**
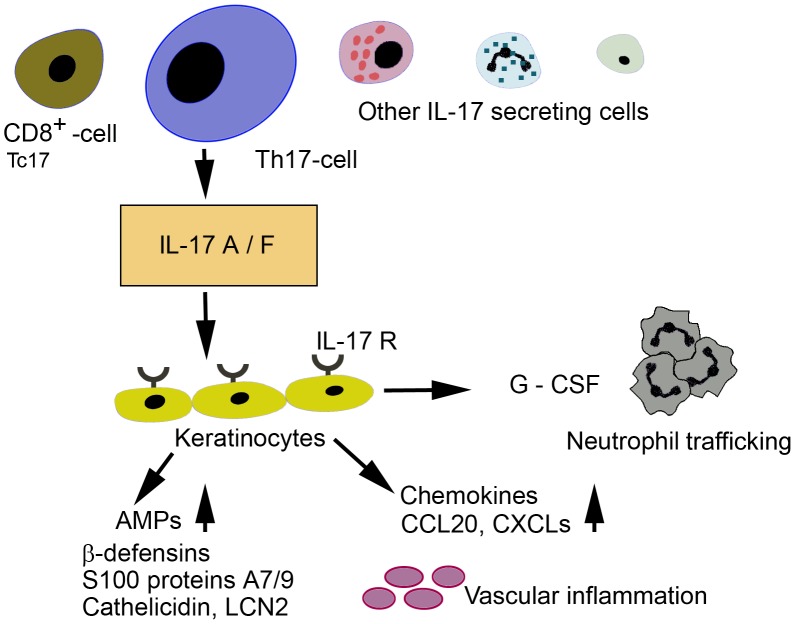
The effect of IL-17 on keratinocytes. Th17-cells are defined to secrete IL-17 cytokines. Also some other cells can secrete IL-17 cytokines when properly activated. IL-17A and IL-17F regulate genes on keratinocytes, that are involved in innate immune defence, such as antimicrobial peptides (beta-defensins, S100 proteins A7/9, cathelicidin, LCN2) and a range of chemokines G-CSF, CXCLs and CCL20 which regulate neutrophil and lymphocyte trafficking. IL-17A and IL-17F stimulate keratinocytes leading to keratinocyte proliferation. Also vascular inflammation is activated.

We found increased expressions of pro-inflammatory cytokines IL-1β, IL-6, TNF-α and TGF-β that are also important in Th17 cell activation. All these cytokines enhance generation of Th17 cells from naive T-cells with activated dendritic cells in the presence of IL-23 [40]. TGF-β is a critical cytokine in both Th17 and Treg-cell differentiation. TNF-α can synergize with IL-17 to promote inflammation in psoriasis and also IL-17 and IFN-γ synergize in pro-inflammatory cytokine production in keratinocytes [49], [50]. On the contrary to Agak et al [20], we found also increased expression of Th17 cells stabilizing cytokine IL-23p19 both at the RNA and protein level in vivo. IL-23 is secreted by activated dendritic cells and macrophages and situations in vitro and in vivo are different. Our results are in line with the prior observations concerning up-regulated TNF-α and IL-1β in acne lesions examined by RT-PCR [25]. Recently *P. acnes* is identified as a trigger of monocyte-macrophage NLRP3- inflammasome activation and IL-1β processing and secretion, and further as a trigger of an innate immune response in the skin [15]. The expression of cytokines IL-6, IL-12p40, INF-γ and TGF-β have not been studied earlier in acne lesions as also noticed by Thiboutot and co-workers [19], nor cytokines IL-17A, IL-17F, IL-23p19, IL-22 and chemokines CCL20 and CSF2. The IL23/Th17 pathway and especially cytokine IL-17A is a key cytokine that activates pathogenic inflammation in psoriasis [51], [52]. We hypothesize that follicular hyperkeratinization, increased expression of AMPs and accumulation of neutrophils in acne lesions may be in part explained by the activation of the IL-23/IL-17 axis (Figure 9).

Previously described CD4+ T cells in early acne lesions have not been determined in details [4], [5]. We defined the markers of T cell subsets at mRNA level and studied Th1 (T-bet), Th17 (IL-17A), Treg (Foxp3) cells, cytotoxic CD8+ T-cells and CD68 macrophages and CD83 positive activated dendritic cells in clinically early acne lesions by immunohistochemistry both phenotypically and proportionally (Figure 5, Table 7). Recently Agak and co-workers [20] presented IL-17+ lymphoid cells in three closed comedone-type acne lesions by immunohistochemistry. In addition to Th17 cells IL-17A can also be secreted by CD8+ cytotoxic T-cells (Tc17), αβ and γδ T cells and even neutrophils and mast cells [53]. In our study the observations of IL-17A+ by immunohistochemistry resembled the findings made by Res et al [54] in psoriasis. The cells were few and they were scattered throughout the samples (Table 7, Figure 5a), however, significantly more numerous in lesional areas. IL-17A+ cells were mainly lymphocytes but some of them were neutrophils and even mast cells. Further investigations are needed to examine whether CD8+ cells in acne lesions were either Tc17 or Tc22 cytotoxic cells. To examine early CD4+ reaction, biopsies were taken from small visible developing papules for microarray/RT-PCR and immunohistochemistry. Previously Norris and Cunliffe have described the biopsied 6-72 h lesions had morphology of small papules with a minimal surrounding erythematous flare, in which lymphoid cells outnumbered polymorphonuclear cells (PMNs) in the 6 h biopsies and PMNs became increasingly numerous in the 24-72 h lesions [5]. In our study the biopsied lesions were similar in appearance: comedones with erythematous flare or small papules (Figure 1). The lesions in immunohistochemistry revealed neutrophils in many of the lesions but fewer than lymphoid cells and moreover the rupture of the follicle walls was not usually seen suggesting the biopsied lesions were not fully developed inflammatory lesions.

To best of our knowledge Treg cells have not been examined in acne lesions earlier. We found significantly increased number of Foxp3+ cells in papillary dermis in immunohistochemistry. Tregs prevent autoimmunity and suppress immune responses. Retinoic acid can regulate reciprocally Tregs and Th17 via TGF-β -dependent generation of Foxp3 [55], [56], a mechanism that may be of importance in the treatment of acne by isotretinoin. Elevated serum levels of anti-inflammatory cytokine IL-10 are found in acne patients and the expression of IL-10 is increased in acne lesions [13], [25]. In our study lesional IL-10 mRNA was higher in acne than in psoriasis (Figure 8). Of note, TLR2 recognition of *Candida albicans* suppresses inflammatory responses via production of IL-10 and enhanced Treg survival [14] but it is not known if TLR2 recognition in acne has the same consequences. Though acne is often a chronic disease, a single acne lesion is seldom secondarily infected and is rapidly demarcated. The increased IL-10 expression and Tregs may demarcate the inflammation of a single acne lesion efficiently from the beginning. The reaction soon changes after ruptures of follicular walls into the direction of wound healing and fibrosis [34].

In conclusion, we demonstrated the presence of IL-17A+ cells, which were mainly lymphocytes, in clinically early visible inflamed acne lesions, and also the activation of cytokines, chemokines and antimicrobial peptides known to be typical for the Th17/IL-17 pathway. Th1, Th17 and also CD8+ activation and IL-17 related AMP and CXCL chemokine production with neutrophil attraction in acne lesions could be important factors among others in acne pathogenesis. Further studies will show the relevance of these findings also in the pathogenesis of different variations of severe acne such as acne cystica, acne fulminans and autoinflammatory syndromes with acne symptoms like SAPHO or PAPA syndromes, and indicate new possibilities to target the inflammatory response in acne diseases.

## Supporting Information

Figure S1 **A flowchart of the experimental design.** The figure clarifies the methods used in the different cohorts of the study.
(TIF)

## References

[1] Knutson DD (1974) Ultrastructural observations in acne vulgaris: The normal sebaceous follicle and acne lesions. J Invest Dermatol 62: 288–307. 4361988 10.1111/1523-1747.ep12676804

[2] Guy R, Kealey T (1998) The effects of inflammatory cytokines on the isolated human sebaceous infundibulum. J Invest Dermatol 110: 410–415. 9540984 10.1046/j.1523-1747.1998.00143.x

[3] Jeremy AH, Holland DB, Roberts SG, Thomson KF, Cunliffe WJ (2003) Inflammatory events are involved in acne lesion initiation. J Invest Dermatol 121: 20–27. 12839559 10.1046/j.1523-1747.2003.12321.x

[4] Layton AM, Morris C, Cunliffe WJ, Ingham E (1998) Immunohistochemical investigation of evolving inflammation in lesions of acne vulgaris. Exp Dermatol 7: 191–197. 9758417 10.1111/j.1600-0625.1998.tb00323.x

[5] Norris JF, Cunliffe WJ (1988) A histological and immunocytochemical study of early acne lesions. Br J Dermatol 118: 651–659. 2969256 10.1111/j.1365-2133.1988.tb02566.x

[6] Kim J, Ochoa MT, Krutzik SR, Takeuchi O, Uematsu S, et al (2002) Activation of toll-like receptor 2 in acne triggers inflammatory cytokine responses. J Immunol 169: 1535–1541. 12133981 10.4049/jimmunol.169.3.1535 PMC4636337

[7] Georgel P, Crozat K, Lauth X, Makrantonaki E, Seltmann H, et al (2005) A toll-like receptor 2-responsive lipid effector pathway protects mammals against skin infections with gram-positive bacteria. Infect Immun 73: 4512–4521. 16040962 10.1128/IAI.73.8.4512-4521.2005 PMC1201198

[8] Jugeau S, Tenaud I, Knol AC, Jarrousse V, Quereux G, et al (2005) Induction of toll-like receptors by Propionibacterium acnes. Br J Dermatol 153: 1105–1113. 16307644 10.1111/j.1365-2133.2005.06933.x

[9] Kim J (2005) Review of the innate immune response in acne. Activation of toll-like receptor 2 in acne triggers inflammatory cytokine responses. Dermatology 211: 193–198. 16205063 10.1159/000087011

[10] Kollisch G, Kalali BN, Voelcker V (2005) Various members of the toll-like receptor family contribute to the innate immune response of human epidermal keratinocytes. Immunology 114: 531–541. 15804290 10.1111/j.1365-2567.2005.02122.x PMC1782101

[11] Nagy I, Pivarcsi A, Koreck A, Széll M, Urbán E, et al (2005) Distinct strains of Propionibacterium acnes induce selective human beta-defensin-2 and interleukin-8 expression in human keratinocytes through toll-like receptors. J Invest Dermatol 124: 931–938. 15854033 10.1111/j.0022-202X.2005.23705.x

[12] Oeff MK, Seltmann H, Hiroi N, Nastos A, Makrantonaki E, et al (2006) Differential regulation of Toll-like receptor and CD14 pathways by retinoids and corticosteroids in human sebocytes. Dermatology 213: 266. 17033190 10.1159/000095056

[13] Dispenza MC, Wolpert EB, Gilliland KL, Dai JP, Cong Z, et al (2012) Systemic isotretinoin therapy normalizes exaggerated TLR-2-mediated innate immune responses in acne patients. J Invest Dermatol 132: 2198–2205. 22513780 10.1038/jid.2012.111 PMC3614089

[14] Hernández-Santos N, Gaffen SL (2012) Th17 cells in immunity to Candida albicans. Cell Host Microbe 11: 425–435. 22607796 10.1016/j.chom.2012.04.008 PMC3358697

[15] Kistowska M, Gehrke S, Jankovic D, Kerl K, Fettelschoss A, et al (2014) IL-1β drives inflammatory responses to Propionibacterium acnes in vitro and in vivo. J Invest Dermatol 134: 677–685. 24157462 10.1038/jid.2013.438

[16] Qin M, Pirouz A, Kim MH, Krutzik SR, Garbán HJ, et al (2014) Propionibacterium acnes induces IL-1β secretion via the NLRP3 inflammasome in human monocytes. J Invest Dermatol 134: 381–388. 23884315 10.1038/jid.2013.309 PMC4116307

[17] Shaheen B, Gonzalez M (2011) A microbial aetiology of acne: what is the evidence? Br J Dermatol 165: 474–485. 21495996 10.1111/j.1365-2133.2011.10375.x

[18] Zouboulis CC, Jourdan E, Picardo M (2014) Acne is an inflammatory disease and alterations of sebum composition initiate acne lesions. J Eur Acad Dermatol Venereol 28: 527–532. 24134468 10.1111/jdv.12298

[19] Thiboutot DM, Layton AM, Eady EA (2014) IL-17: a key player in the P. acnes inflammatory cascade? J Invest Dermatol 134: 307–310. 24424453 10.1038/jid.2013.400

[20] Agak GW, Qin M, Nobe J, Kim MH, Krutzik SR, et al (2014) Propionibacterium acnes induces an interleukin-17 response in acne vulgaris that is regulated by vitamin A and vitamin D. J Invest Dermato 134: 366–373. 10.1038/jid.2013.334 PMC4084940 23924903

[21] Furusawa H, Suzuki Y, Miyazaki Y, Inase N, Eishi Y (2012) Th1 and Th17 immune responses to viable Propionibacterium acnes in patients with sarcoidosis. Respir Investig 50: 104–109. 10.1016/j.resinv.2012.07.001 23021769

[22] Sugisaki H, Yamanaka K, Kakeda M, Kitagawa H, Tanaka K, et al (2009) Increased interferon-gamma, interleukin-12p40 and IL-8 production in Propionibacterium acnes-treated peripheral blood mononuclear cells from patient with acne vulgaris: host response but not bacterial species is the determinant factor of the disease. J Dermatol Sci 55: 47–52. 19375895 10.1016/j.jdermsci.2009.02.015

[23] Chronnell CM, Ghali LR, Ali RS, Quinn AG, Holland DB, et al (2001) Human beta defensin -1 and -2 expression in human pilosebaceous units: Up-regulation in acne vulgaris lesions. J Invest Dermatol 117: 1120–1125. 11710922 10.1046/j.0022-202x.2001.01569.x

[24] Ingham E, Eady EA, Goodwin CE, Cove JH, Cunliffe WJ (1992) Pro-inflammatory levels of interleukin-1 alpha-like bioactivity are present in the majority of open comedones in acne vulgaris. J Invest Dermatol 98: 895–901. 1534342 10.1111/1523-1747.ep12460324

[25] Kang S, Cho S, Chung JH, Hammerberg C, Fisher GJ, et al (2005) Inflammation and extracellular matrix degradation mediated by activated transcription factors nuclear factor-kappaB and activator protein-1 in inflammatory acne lesions in vivo. Am J Pathol 166: 1691–1699. 15920154 10.1016/s0002-9440(10)62479-0 PMC1602424

[26] Trivedi NR, Gilliland KL, Zhao W, Liu W, Thiboutot DM (2006) Gene array expression profiling in acne lesions reveals marked upregulation of genes involved in inflammation and matrix remodeling. J Invest Dermatol 126: 1071–1079. 16528362 10.1038/sj.jid.5700213

[27] Bolstad BM, Irizarry RA, Astrand M, Speed TP (2003) A comparison of normalization methods for high density oligonucleotide array data based on bias and variance. Bioinformatics 19: 185–193. 12538238 10.1093/bioinformatics/19.2.185

[28] Benjamini Y, Hochberg Y (1995) Controlling the false discovery rate: a practical and powerful approach to multiple testing. J Roy Statist Soc Ser B (Methodological) 57: 289–300.

[29] Lehtimäki S, Savinko T, Lahl K, Sparwasser T, Wolff H, et al (2012) The temporal and spatial dynamics of Foxp3+ Treg cell-mediated suppression during contact hypersensitivity responses in a murine model. J Invest Dermatol 132: 2744–2751. 22739792 10.1038/jid.2012.212

[30] Väyrynen JP, Vornanen JO, Sajanti S, Böhm JP, Tuomisto A, et al (2012) An improved image analysis method for cell counting lends credibility to the prognostic significance of T cells in colorectal cancer. Virchows Arch 460: 455–465. 22527018 10.1007/s00428-012-1232-0

[31] Edgar R, Domrachev M, Lash AE (2002) Gene Expression Omnibus: NCBI gene expression and hybridization array data repository. Nucleic Acids Res 30: 207–210. 11752295 10.1093/nar/30.1.207 PMC99122

[32] Fogel P, Young SS, Hawkins DM, Ledirac N (2007) Inferential, robust non-negative matrix factorization analysis of microarray data. Bioinformatics 23: 44–49. 17092989 10.1093/bioinformatics/btl550

[33] Alestas T, Ganceviciene R, Fimmel S, Müller-Decker K, Zouboulis CC (2006) Enzymes involved in the biosynthesis of leukotriene B4 and prostaglandin E2 are active in sebaceous glands. J Mol Med (Berl) 84: 75–87. 16388388 10.1007/s00109-005-0715-8

[34] Kurokawa I, Danby FW, Ju Q, Wang X, Xiang LF, et al (2009) New developments in our understanding of acne pathogenesis and treatment. Exp Dermatol 18: 821–832. 19555434 10.1111/j.1600-0625.2009.00890.x

[35] Nagy I, Pivarcsi A, Kis K, Koreck A, Bodai L, et al (2006) Propionibacterium acnes and lipopolysaccharide induce the expression of antimicrobial peptides and proinflammatory cytokines/chemokines in human sebocytes. Microbes Infect 8: 2195–2205. 16797202 10.1016/j.micinf.2006.04.001

[36] Begon E, Michel L, Flageul B, Beaudoin I, Jean-Louis F, et al (2007) Expression, subcellular localization and cytokinic modulation of toll-like receptors (TLRs) in normal human keratinocytes: TLR2 up-regulation in psoriatic skin. Eur J Dermatol 17: 497–506. 17951129 10.1684/ejd.2007.0264

[37] Bangert C, Brunner PM, Stingl G (2011) Immune functions of the skin. Clin Dermatol 29: 360–376. 21679864 10.1016/j.clindermatol.2011.01.006

[38] Mouser PE, Baker BS, Seaton ED, Chu AC (2003) Propionibacterium acnes-reactive T helper-1 cells in the skin of patients with acne vulgaris. J Invest Dermatol 121: 1226–1228. 14708633 10.1046/j.1523-1747.2003.12550_6.x

[39] Loser K, Beissert S (2012) Regulatory T cells: banned cells for decades. J Invest Dermatol 132: 864–871. 22158548 10.1038/jid.2011.375

[40] Maddur MS, Miossec P, Kaveri SV, Bayry J (2012) Th17 cells: Biology, pathogenesis of autoimmune and inflammatory diseases, and therapeutic strategies. Am J Pathol 181: 8–18. 22640807 10.1016/j.ajpath.2012.03.044

[41] Martin DA, Towne JE, Kricorian G, Klekotka P, Gudjonsson JE, et al (2013) The emerging role of IL-17 in the pathogenesis of psoriasis: Preclinical and clinical findings. J Invest Dermatol 133: 17–26. 22673731 10.1038/jid.2012.194 PMC3568997

[42] Sabat R, Wolk K (2011) Research in practice: IL-22 and IL-20: Significance for epithelial homeostasis and psoriasis pathogenesis. J Dtsch Dermatol Ges 9: 518–523. 21251229 10.1111/j.1610-0387.2011.07611.x

[43] Wolk K, Warszawska K, Hoeflich C, Witte E, Schneider-Burrus S, et al (2011) Deficiency of IL-22 contributes to a chronic inflammatory disease: pathogenetic mechanisms in acne inversa. J Immunol 186: 1228–1239. 21148041 10.4049/jimmunol.0903907

[44] Liang SC, Tan XY, Luxenberg DP, Karim R, Dunussi-Joannopoulos K, et al (2006) Interleukin (IL)-22 and IL-17 are coexpressed by Th17 cells and cooperatively enhance expression of antimicrobial peptides. J Exp Med 203: 2271–2279. 16982811 10.1084/jem.20061308 PMC2118116

[45] Ganceviciene R, Fimmel S, Glass E, Zouboulis CC (2006) Psoriasin and follicular hyperkeratinization in acne comedones. Dermatology 213: 270–272. 17033192 10.1159/000095058

[46] Lee DY, Yamasaki K, Rudsil J, Zouboulis CC, Park GT, et al (2008) Sebocytes express functional cathelicidin antimicrobial peptides and can act to kill Propionibacterium acnes. J Invest Dermatol 128: 1863–1866. 18200058 10.1038/sj.jid.5701235 PMC2632971

[47] Lumsden KR, Nelson AM, Dispenza MC, Gilliland KL, Cong Z, et al (2011) Isotretinoin increases skin-surface levels of neutrophil gelatinase-associated lipocalin in patients treated for severe acne. Br J Dermatol 165: 302–310. 21466536 10.1111/j.1365-2133.2011.10362.x PMC3142306

[48] Nelson AM, Zhao W, Gilliland KL, Zaenglein AL, Liu W, et al (2008) Neutrophil gelatinase-associated lipocalin mediates 13-cis retinoic acid-induced apoptosis of human sebaceous gland cells. J Clin Invest 118: 1468–1478. 18317594 10.1172/JCI33869 PMC2262030

[49] Chiricozzi A, Guttman-Yassky E, Suárez-Fariñas M, Nograles KE, Tian S, et al (2011) Integrative responses to IL-17 and TNF-α in human keratinocytes account for key inflammatory pathogenic circuits in psoriasis. J Invest Dermatol 131: 677–687. 21085185 10.1038/jid.2010.340

[50] Teunissen MB, Koomen CW, de Waal Malefyt R, Wierenga EA, Bos JD (1998) Interleukin-17 and interferon-gamma synergize in the enhancement of proinflammatory cytokine production by human keratinocytes. J Invest Dermatol 111: 645–649. 9764847 10.1046/j.1523-1747.1998.00347.x

[51] Di Cesare A, Di Meglio P, Nestle FO (2009) The IL-23/Th17 axis in the immunopathogenesis of psoriasis. J Invest Dermatol 129: 1339–1350. 19322214 10.1038/jid.2009.59

[52] Krueger JG, Fretzin S, Suárez-Fariñas M, Haslett PA, Phipps KM, et al (2012) IL-17A is essential for cell activation and inflammatory gene circuits in subjects with psoriasis. J Allergy Clin Immunol 130 145–154: e9. 10.1016/j.jaci.2012.04.024 PMC3470466 22677045

[53] Lowes MA, Russell CB, Martin DA, Towne JE, Krueger JG (2013) The IL-23/T17 pathogenic axis in psoriasis is amplified by keratinocyte responses. Trends Immunol 34: 174–181. 23291100 10.1016/j.it.2012.11.005 PMC3721313

[54] Res PC, Piskin G, de Boer OJ, van der Loos CM, Teeling P, et al (2010) Overrepresentation of IL-17A and IL-22 producing CD8 T cells in lesional skin suggests their involvement in the pathogenesis of psoriasis. PLoS One 5: e14108. 21124836 10.1371/journal.pone.0014108 PMC2991333

[55] Elias KM, Laurence A, Davidson TS, Stephens G, Kanno Y, et al (2008) Retinoic acid inhibits Th17 polarization and enhances FoxP3 expression through a Stat-3/Stat-5 independent signaling pathway. Blood 111: 1013–1020. 17951529 10.1182/blood-2007-06-096438 PMC2214761

[56] Xiao S, Jin H, Korn T, Liu SM, Oukka M, et al (2008) Retinoic acid increases Foxp3+ regulatory T cells and inhibits development of Th17 cells by enhancing TGF-beta-driven Smad3 signaling and inhibiting IL-6 and IL-23 receptor expression. J Immunol 181: 2277–2284. 18684916 10.4049/jimmunol.181.4.2277 PMC2722959

