# Supplementary figures and images for: IL-17/Th17 Pathway Is Activated in Acne Lesions
Source: PLoS One. 2014 Aug 25;9(8):e105238. doi: 10.1371/journal.pone.0105238 (PMC4143215; doi:10.1371/journal.pone.0105238)

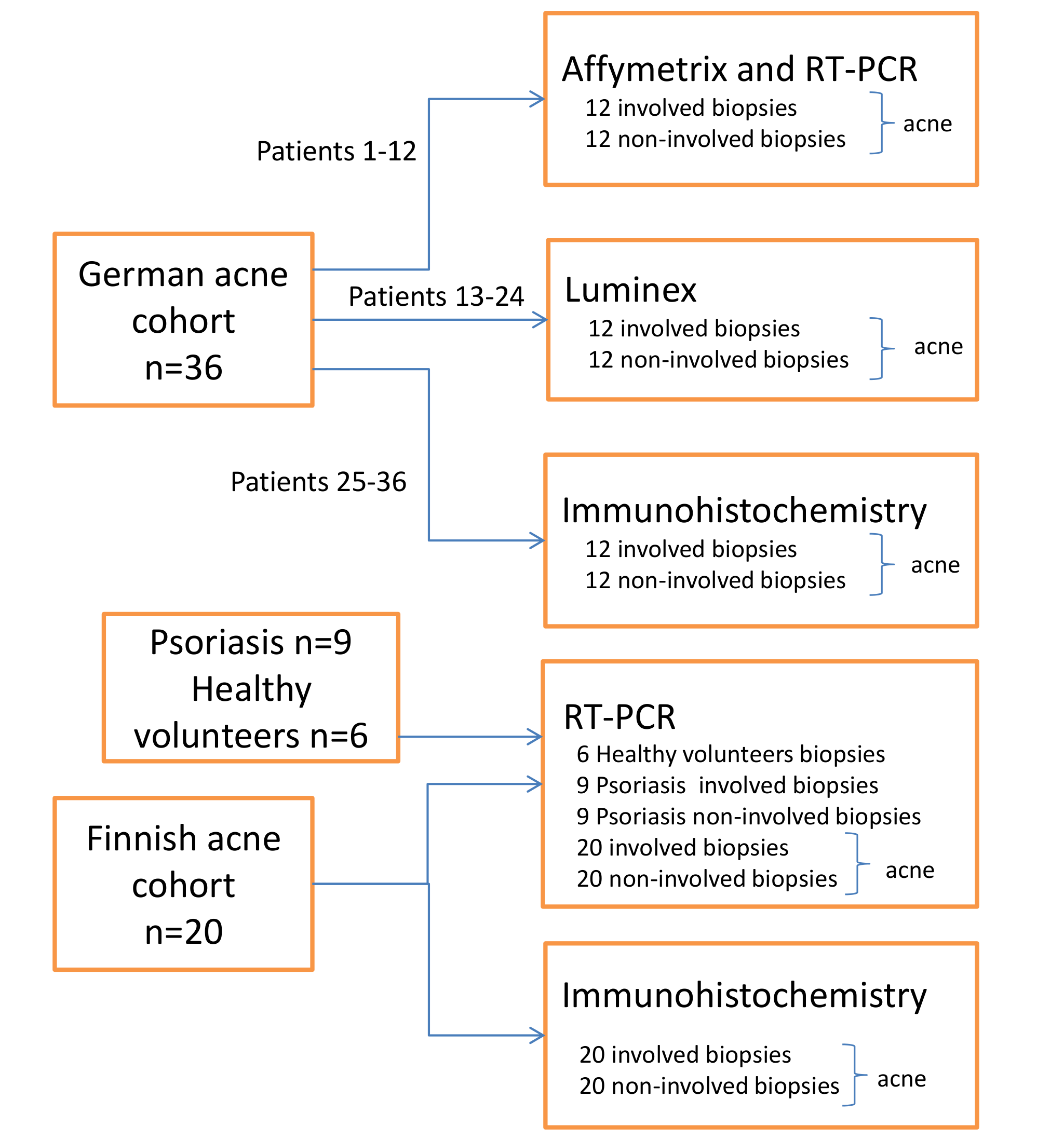

Supplement: Figure S1 — A flowchart of the experimental design. The figure clarifies the methods used in the different cohorts of the study. (TIF) [file pone.0105238.s001.tif]
